# Trends in the discussion of cycling in urban environments: An X-based study

**DOI:** 10.1371/journal.pone.0330616

**Published:** 2025-11-18

**Authors:** Laura Antón-González, Maite Pellicer-Chenoll, Israel Villarrasa-Sapiña, José-Luis Toca-Herrera, Luis-Millán González, José Devís-Devís

**Affiliations:** 1 Departament Didàctica i Organització Escolar, Facultat de Filosofia i Ciències de l’Educació, Universitat de València, València, Spain; 2 Departament d’Educació Física i Esportiva, Universitat de València, València, Spain; 3 Department of Bionanosciences, Institute of Biophysics, University of Natural Resources and Life Sciences Vienna (BOKU), Vienna, Austria; CCET: Chandigarh College of Engineering and Technology, INDIA

## Abstract

Urban transportation addresses issues affecting the population and environment of a city. To handle this problem, scientific literature and public policies are increasingly oriented towards promoting sustainable means of transport in urban environments, such as cycling. Public opinion is a key factor in shaping these policies for greater effectiveness and, therefore, must be taken into consideration. This study aims to analyse the topics and sentiments expressed by bicycle users on X (formerly Twitter) regarding urban cycling since the implementation of the Sustainable Development Goals (SDGs). Posts published between 2016 and 2022 were retrieved using the X search API and processed through text mining techniques to identify the main themes and emotional tones. The results reveal a predominance of positive perceptions towards cycling, often linked to its benefits for health, mobility, and sustainability. At the same time, recurring negative sentiments reflect user concerns about infrastructure limitations and coexistence with motor vehicles. These dual narratives suggest that, while cycling is widely recognised as a valuable means of urban transport, its development still faces significant challenges in practice. In conclusion, the analysis of public discourse on social media provides valuable insights into how cycling is perceived and discussed by urban residents. These findings underline the potential of platforms like X to inform the development of more inclusive and effective mobility policies. By aligning policy interventions with citizen perspectives, such strategies can foster greater public engagement and contribute more effectively to the achievement of the SDGs.

## Introduction

Urban populations and their environments are currently affected by various problems related to the use of motorised means of transport [1]. Therefore, in recent years, there have been concerns about promoting environmentally friendly means of transport around cities that also offer benefits to the population. Among the various alternatives, bicycles are the most sustainable option for the future [2]. Cycling as a means of urban transport offers various benefits for public health and the environment. In the health field, urban cycling provides benefits which include improved cardiorespiratory fitness, reduced risk factors for disease, and significantly reduced risk of mortality from cancer, cardiovascular disease, and obesity [3]. Even e-bikes have already demonstrated their positive health effects, including enhancements in mental and physical health, happiness and overall well-being [4], while also enabling people to cycle longer distances compared to conventional bicycles [5]. The use of bicycles as a means of urban transport reduces the use of energy sources and reduces pollution [6]. Furthermore, by substituting motor vehicle usage with cycling, adverse impacts, such as traffic accidents and greenhouse gas emissions, are mitigated [7].

Owing to the growing concern about transforming cities into sustainable environments, the scientific community is focused on highlighting the multiple benefits of cycling. Publications on this subject have increased exponentially in recent decades [8]. However, despite cycling is an active and environmentally friendly option for short and medium distances, it represents a low percentage of journeys in comparison to other means of transport in different cities [9]. To enhance the use of cycling, it would be beneficial to implement feedback systems that can identify areas for improvement in active transportation. In this sense, knowledge gained through social media can be utilised to discover and expose users’ preferences in a way that is useful for decision-making in urban planning [10]. The enormous amount of available information does not allow conclusions to be drawn on this issue, making it impossible to deduce the meaning of bicycle users’ opinions in the city. However, advanced text analysis methods like text mining enable the extraction of implicit information from textual data [11]. Even, language models and prompting techniques have also been developed to classify perceptions specifically about cyclists [12]. However, text mining enhances political deliberation among citizens, thus expanding their democratic influence, because text mining techniques allow policymakers to process masses of messages in the policy space or on social media [13]. These techniques have been widely used in this field of knowledge [14–17].

For this purpose, X (formerly Twitter) was selected as a source of data because it provides free and accessible information [18]. This is the most popular microblogging social media platform on which people express their daily activities and concerns [19]. Additionally, the sentiment analysis of X allows us to classify the polarity of sentiments into negative, neutral, and positive [20]. Analysing the polarities of X users’ sentiments has enabled the development of strategies for political elections [21]. Companies also use X-sentiment analysis as an effective way to understand people’s sentiments towards their products and brands [22]. Furthermore, one of the advantages of using X to analyse urban cycling is its ability to geolocate posts, facilitating the association of sentiment with specific populations. Indeed, the analysis of Twitter posts is widely applied in various areas of transport research, including traffic analysis. [23]. Moreover, emojis have been included in the analysis as part of the study because of their communicative functions, such as expressing emotions [24], strengthening expression and adjusting tone [25]. Additionally, to complete our analysis, hashtags were used as the objects of the study. A ‘hashtag’ is a word or a phrase without spaces prefixed with the hash symbol # inserted anywhere in the body of posts [26]. These hashtags allow posts to be framed according to what they mean, allowing users to indicate the specific meaning of what they are posting about [27].

Literature analysing motivation towards cycling in urban environments is abundant. For example, various studies, including the one conducted by Felix et al. [28] and Hook et. al [29], investigate the motivations for cycling through the use of questionnaires. Other studies employ standardized indices to analyses cycling and its relationship with infrastructure [30]. Some even apply text mining techniques to examine scientific articles [31]. However, fewer articles use text mining techniques in social media [14]. Owing to X’s potential for mining, a study was conducted to analyse X users’ feelings about cycling between 2009 and 2016 to extract patterns to promote cycling [15]. The results of this study showed that cycling in a city is associated with weather and seasonal patterns. Furthermore, in terms of the polarity of feelings, the general feeling about cycling has been positive in recent years. However, negative feelings are associated with bad weather, crime, and other problems. A similar study found a generally positive sentiment towards Washington DC’s bike sharing system [14]. Nevertheless, since 2016, there have been substantial changes in policies promoting urban cycling, which may have changed people’s sentiments towards cycling. On 25 September 2015 the United Nations General Assembly approved the 2030 Agenda for Sustainable Development [32]. It contains a set of 17 Sustainable Development Goals (SDG) addressing major global challenges, such as climate change and sustainable cities. Goal 11 (sustainable cities and communities) was developed to transform cities and human settlements into inclusive, safe, resilient, and sustainable environments. Therefore, the use of sustainable and environment-friendly means of transport plays a key role in achieving this goal. In response, governments have taken measures to promote cycling among the population to mitigate the impact of motor vehicles and complement it with other transport policies, turning cities into healthy and sustainable environments.

In this context, the general purpose of our study is to explore how public opinion regarding the use of bicycles as a form of urban transportation is expressed on X. Specifically, the primary objective of our study is to collect and quantify the available content related to bicycles on X (formerly Twitter). As a secondary objective, we aim to analyse the positive and negative emotions expressed by users in posts about cycling. This study is informed by several practical questions our work seeks to address: What emotions are most prevalent in social media posts about urban cycling during the period between 2016 and 2022? What are the dominant topics and key terms associated with cycling on social platforms? And how are social attitudes towards cycling represented through hashtags and emojis?

This study ultimately provides descriptive data that enable the identification of the main ideas expressed in X, as well as a structured overview of the topics that trigger positive and negative user responses. We consider that this approach may offer a useful foundation for professionals involved in the development of urban mobility policies. At the same time, the results may serve as a starting point for further research—whether experimental or qualitative—aimed at improving specific aspects such as cycling infrastructure, incentive programs for active transport, or awareness campaigns.

## Materials and methods

This study adopts an exploratory and descriptive design, primarily based on the quantitative analysis of X data related to urban cycling. Although the methodological approach is predominantly quantitative, qualitative interpretations are incorporated to enhance the contextual understanding of the social meanings embedded in the discourse [33].

To address the research questions, we employed a set of established text mining techniques commonly used in computational social science. The analytical strategy comprised three main components. First, lexical frequency analysis (n-grams) was conducted to identify the most frequently used words, hashtags, and emojis across the dataset [34,35]. Second, sentiment analysis algorithms were applied to detect the emotional polarity of posts, classifying them as positive, neutral, or negative [36]. Third, the dataset was segmented into two major sentiment-based groups—positive and negative—and subjected to topic modelling procedures in order to uncover the dominant thematic structures within each sentiment category [37–39].

This multi-level approach allowed for both the quantification of discourse patterns and the interpretation of their social significance, thereby offering a comprehensive view of how urban cycling is represented on social media.

### Data retrieval and pre-processing

To build the database, a MATLAB script (R2019b, MathWorks Inc., Natick, MA, USA) was used to perform queries using the Twitter search API (“X” since July 2023). The database was accessed using a Twitter Academic Research account (free of charge), which allowed access to the historical archive of tweets published since the creation of the social network (i.e., since 2006). This type of account is specifically intended for non-commercial academic research. To obtain this access, a special request must be submitted detailing the analysis protocols, ensuring they meet ethical research standards and protect user confidentiality.

The search focused on retrieving posts in English containing the term ‘bicycle’ and ‘city’, along with their variations (e.g., ‘bike’ and ‘urban’), combined with the emoji of the urban bicycle or urban cyclists (e.g., 🚲 and 🚴) from 2016 to 2022. Combining emojis and keywords in the search equation will allow us to further adjust the search to our purpose and avoid noise in the analysis.

Conversations associated with the original posts in which users expressed their thoughts about the original post were also retrieved. Published posts were discarded from the search because we focused only on the opinions of the original posts.

The data for each post were stored in JSON format to extract the fields of our analysis. The files contain information on the retrieved post and its creator, encompassing more than 150 attributes, of which we used only 11.

The search was performed on 3 April 2023. About 179, 691 posts were retrieved before removing duplicates, replicas, and reposts. The collection of documents (retrieved posts) is referred to as a corpus.

For data pre-processing, all duplicate posts were removed using the unique post identifier (ID) field. Standard recommendations used in similar studies [40] were followed to prepare post-texts for further analysis. The documents are reduced to tokens (tokenisation), and the following actions are performed:

i All hyperlinks (‘http://url’), hashtags (‘# hashtag’), emojis, and username links (‘@username’) that appeared in the posts were removed
ii Punctuation marks and special characters were removed
iii Words were converted to lowercase
iv Words that could add noise to the text and that did not add content to the posts (e.g., ‘a’, ‘and’, and ‘to’) were removed using a generic stopword list
v The words were standardised through a lemmatisation process, in which a morphological analysis was carried out to reduce them to their roots. In this process, a predefined dictionary is used. To improve this process, part-of-speech details were added to indicate whether the word was a noun, verb, or adjective
vi Words with fewer than two or over 20 characters and whose frequency in the corpus of documents was less than two, were also deleted.

For all the processes described above, the functions implemented in MATLAB’s Text Analytics Toolbox (version 1.4) were used.

From the resulting tokens, one bag of words (unigrams: one token) and two bags of grams (bigrams: two tokens in a row; trigrams: three tokens in a row) were formed.

Original documents (raw data) and associated fields were stored for further analysis. The remaining fields containing text (e.g., username, entity mention, and location) were not pre-processed. Although duplicates were located through their unique identifiers (ID), several posts were published with slight variations from others. Therefore, after the documents were cleaned, posts with slight variations from the original text were removed.

The post IDs used in the analysis were retrieved from the Data S1_id. To comply with X’s terms of use, we only published the collected post-IDs for non-commercial use in this research. Users who want to reuse their IDs may retrieve the original data using existing software on the market (e.g., Hydrator). Given that the retrieved data are public and several users may be unaware of their relevance or further use, we followed ethical recommendations [41].

### Descriptive analysis and dynamics across the years of posts

Descriptive analysis was used to establish the most frequent n-grams in the posts. An n-gram is a sub-sequence of n elements in a given word sequence. For this purpose, the bags of words (including unigrams, bigrams, and trigrams) that were saved after preprocessing and generated from the posts were employed. The country of origin was identified to determine the locations of X users. To accomplish this, the country of origin was identified for each user by examining the location field in their profile. Ultimately, 10 564 locations were identified.

To comprehend the dynamics across the years included in the study period, we elaborated on a figure to establish the growth and decline in the frequency of posts related to urban cycling.

### Emojis

Emojis, defined as a standardised set of small pictorial glyphs representing everything from smiley faces to international flags, have witnessed an exponential increase in use on social media [42]. Therefore, including them in the analysis is essential to get closer to the communicative reality in social networks. To perform sentiment analysis of the posts, they were first extracted from the text of the posts and then stored separately. Subsequently, all of them were counted, and the most repeated ones were graphically represented (using the Symbola.ttf font for Unicode symbols). In addition, the most common meanings were retrieved using the Full Emoji List [43]. Emoticons were not included in the analyses to facilitate text pre-processing (i.e., removal of punctuation marks and special characters).

### Sentiment analysis

The preprocessing for emotion analysis was slightly different from that used for the description of the tweets. Although a generic stopword list was used, some words that are usually removed were retained, such as certain negations (“not”) or intensity modifiers (“very”). This approach is used because, although these words do not carry meaning on their own, they do influence the polarity of sentiments. Emojis were also included, as they often emphasize the content and, consequently, the positive or negative message being conveyed [44].

Sentiment analysis is a technique that analyses the sentiments in written language This method assigns a combination of words in a text, such as positive, neutral, or negative words, to a sentiment. Therefore, we obtained a score for the frequencies of positive and negative terms. First, a polarity score was calculated for each post, which is defined as the algebraic sum of terms classified as positive or negative divided by the total number of words in the post (Hu and Liu, 2004). Then, posts are classified as ‘positive’ (score > 0), ‘neutral’ (score = 0) or ‘negative’ (score < 0). Modifier words such as negators (e.g., not), amplifiers (e.g., very), and de-amplifiers (e.g., barely) were also considered in the estimation. In this study, we used the VADER algorithm implemented in the MATLAB environment. After assigning a polarity score to each post, to ensure post polarity, posts were divided into very positive (i.e., score > 0.2) and very negative (i.e., score <−0.2). Subsequently, the classified posts were extensively read to verify the content.

### Topic modelling and posts classification

In order to discover the topics collected in the corpus (i.e., collection of posts), a Latent Dirichlet Allocation (LDA) model was used [45]. This model assumes the existence of a fixed number of latent topics that span across multiple documents (i.e., *n* downloaded posts). Every document is defined by a fusion of themes, with each theme being defined by a discrete probability distribution across words. In other words, the probability of a specific word occurring in a textual document is contingent upon the presence of a latent topic [46]. In this study, the LDA model served a dual purpose: 1) to extract the main topics from positive and negative posts, and 2) to function as a method for selecting posts related to certain topics of interest.

For the analysis, we employed a function integrated in MATLAB text analytics toolbox. We used the previously pre-processed bag-of-words (unigram). Initially, we ensured strong coherence among the resultant topics to determine the necessary number of topics. To ensure the adequacy of the LDA model, the perplexity, which indicates how accurately the model describes a collection of documents, was computed. Adjustments were undertaken for 5, 10, 15, 20, 25, and 30 topics. Once the number of topics was established, an LDA model using the Gibbs sampling algorithm was executed [47].

In the present study, the 10-topic model achieved the best fit. For each topic, we selected the top ten words with the highest likelihood of being associated with that particular topic. The probabilities of observing each word in each topic of an LDA model are referred to as topic word probabilities. Additionally, we computed the topic mixtures of the gathered documents to identify the most representative posts for each selected topic. This calculation is used to identify the most representative posts for each topic.

The LDA model not only identify the key topics but also facilitates the unsupervised classification of closely associated posts. In general, for a post to be classified under a topic, it must achieve a probability equal to or greater than 0.6. of belonging to that topic. In this context, we limit the number of posts that best represent a topic. These posts form the basis for discussing the identified topics. The analysis of these posts followed a procedure similar to that of a systematic review. Initially, members of the research group read these posts, emphasising the qualitative aspects of interest in each selected post. Subsequently, the most significant aspects of each topic were shared to build a discussion for this article.

## Evaluation of results, discussion and limitations

### General user data and posts retrieved

**Fig 1:**
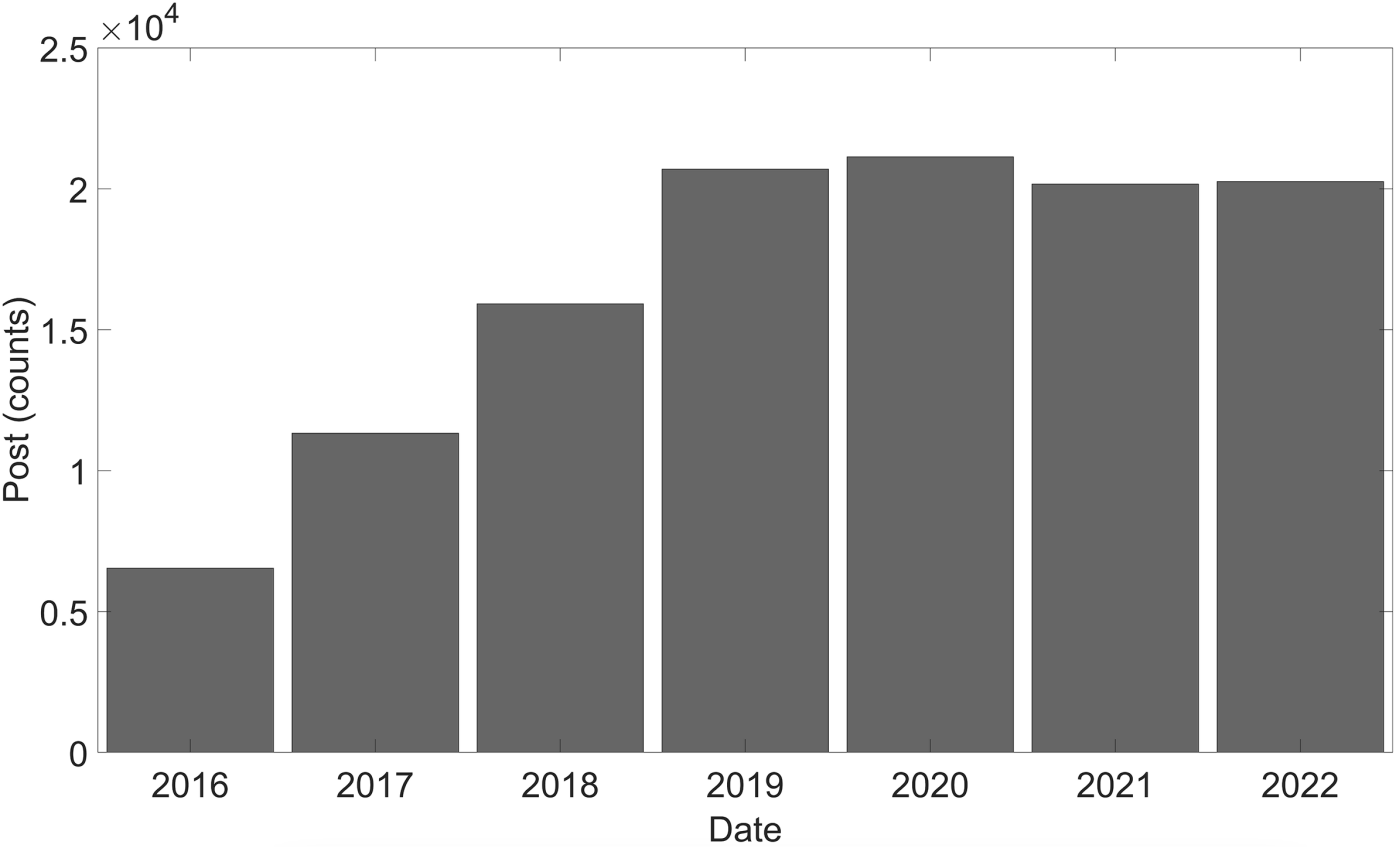
Number of posts related to bike and city during the studied period.

After pre-processing, 116,051 posts were analysed (see S1 Appendix). The first post included in the analysis was published in 2016. From the first post, exponential growth can be observed until 2020, after which it shows a slight decline with subsequent stabilisation (see Fig 1). However, other studies have found a continuous exponential increase in the number of posts related to cycling promotion [15]. Moreover, not only the number of posts related to the topic is increasing, but also the scientific production [48]. This shows that it is an issue that concerns both the scientific community and the urban population. This growth in posts can be attributed to the increased prevalence of cycling as a means of transport in cities, as policies are oriented towards promoting active and sustainable transport [49]. Cycling accounts for a greater percentage than any other means of urban transportation [50].

Table 1 presents the statistical data detailing the number of reposts, replies, and likes garnered from the posts.

**Table 1:** Statistical data on the number of reposts, replies and likes.

*Total posts Reposts (count)*	315 057
never reposted (%)	58.46
reposted >10 times (%)	4.32
mean reposted (reposts/posts)	2.71
***Total posts Replies (count)***	80 416
never replies (%)	74.41
replies >10 replies (%)	0.84
mean replies (replies/posts)	0.69
***Total posts Likes (count)***	1 365 074
never like (%)	30.17
like >10 like (%)	16.74
mean (like/posts)	11.76

**Fig 2:**
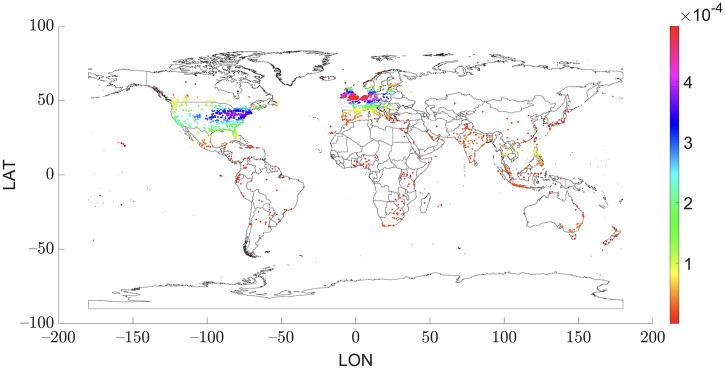
Word clouds of the most frequent words and hashtags found in posts. A larger size represents a higher probability of appearing in the posts and a smaller size represents a lower probability of appearing in the posts. To review terms with lower frequencies of appearance, readers can refer to S2 Appendix.

A total of 9,641 (unique) words observed in all the posts analysed. Among theses, the most frequently repeated words were ‘bike’, ‘city’, and ‘cycle’, which were part of the search strategy used. However, one of the aims of this study was to identify the key terms that appeared alongside the most frequently used ones. Fig 2 shows the word clouds of the most repeated unigrams, bigrams, trigrams, and hashtags in our document.

The most frequent unigrams, excluding those used in the search equation and accompanying verbs or prepositions, were ‘good’, ‘new’, ‘day’, and ‘great’. Among the bigrams, ‘city council’, ‘bike ride’, and ‘bike lane’ were the most repeated and ‘good city will’ was the most prominent trigram.

As for the most recurrent unigrams, we have found words with positive connotations such as ‘good’ or ‘great’. These words were among the most frequently mentioned in previous studies [15]. This is in line with studies that analyse cycling as a means of transport from a qualitative perspective, being perceived as good, guaranteeing physical activity and higher levels of psychological well-being, as well as environmental benefits [51]. Therefore, several posts have included positive aspects related to cycling in urban environments, such as

It's been a while! Just a wee dam run and round the village, good to be back on the bike! 🤟 🚴 ♀ https://t.co/oj5ZoJYWph

Great to see so many people cycling around the city this morning! 🚴 ♀🚴 ♂ 🚵

Cycling more isn't just good for the environment - it's great for our health and the economy too 🚲 🙌 [...]

Regarding bigram, ‘bike lane’ was the recurring theme among bicycle users, referring to cycling infrastructure. To improve the cycling experience and encourage people to cycle more, it is important to build safe cycling lanes [52]. Notably, among the different systems that analyse and improve their quality [30], the consideration of public opinion on the subject is emphasised. It is essential to analyse what users say about cycle lanes because there is often an information gap between politicians and cyclists. Although decision-makers believe that main roads with cycling infrastructure are decisive for fast and safe cycling, cyclists also emphasise the positive effects of green spaces, the experience of the natural environment, and the healthy and recreational effects of cycling [53]. In this paper, cyclists reported that they prefer to take secondary roads and are willing to take detours to integrate with the natural environment and avoid traffic jams, noise, and air pollution — aspects rarely acknowledged by decision-makers, for example:

Yes to Trees 🌳, Yes to Protected Bike Lanes, 🚲 💪 Yes to Pedestrian Sidewalks, 🚶 ♀ Yes to Sustainable Cities, Yes to people-friendly Cities. @NMS_Kenya @KURAroads @NziokaWaita @PSCharlesHinga @Ma3Route @KenyanTraffic https://t.co/kuQkh1YNDM

Curiously, the first 29 trigrams were related to an electoral campaign of the ‘Boulder Progress’ political group (Colorado, United States), which strongly emphasised the promotion of bicycles as a means of transport from a climate perspective. These results show that X is a space where people discuss political issues, including cycling, in urban environments. Indeed, owing to climate emergencies, several political groups are increasingly recognising the need to redesign and rebuild cities for sustainable transport [54]. Thus, when implementing a cycling campaign, X can be an effective tool for obtaining direct user feedback. Apart from this, the trigrams that have appeared most frequently are ‘New York City’ and ‘protect bike lane’. Despite being a city with the potential to emerge as a leading city for cycling because of its flat terrain and proximity to destinations, New York faces various challenges. Issues such as heavy traffic, poor pavement conditions, poor bridge connections, vehicle exhaust fumes, lack of safe bicycle parking and theft act as deterrents to cycling [55], and they also provoke discussions on X. Regarding trigram ‘protect bike lane’, safety has been a recurring theme among users, mentioning the need to build protected cycle lanes. Cycling close to motor traffic was one of the main barriers for 1,079 citizens (cycling and noncycling populations) in Lisbon (Portugal) [28]. Therefore, the removal of this barrier could enable cycling. Below are some of the user comments related to the protection of cycle lanes, as an example:

More bike lanes 🚲 = less traffic 🚗. More bike lanes=safer streets. This isn't new information, if want safer, cleaner, and healthier cities, we need to invest in bike infrastructure https://t.co/ey1R2QYJnf

🚲 @voxdotcom - By adding protected bike lines — separated from car lanes by a barrier for safety — biking becomes a safe, accessible alternative to shorter drives. https://t.co/YwygOh44v1 #82alliance https://t.co/d1n3bRPD4h

As for hashtags, the most repeated ones were #cycling (4,126) and #bike (1,515). These two hashtags show that the most frequent use of hashtags is to report where they are cycling in a city. Moreover, we have found #mobility (904) among the most mentioned hashtags, which corroborates the importance of cycling as a mobility alternative in the urban environment. Another important hashtag is WorldBicycleDay (610), indicating that X is a space to reach out to the population to promote the use of bicycles at this type of festivity.

Additionally, among the most repeated hashtags was #COVID19 (460). This indicates that cycling was a topic of discussion among the issues raised by urban cyclists during the pandemic. Studies on Twitter and COVID-19 reached some very interesting conclusions. On one hand, they established how the pandemic affected mobility and the reallocation of urban spaces. The results indicate that, due to the risk of contagion, many people avoided public transportation, opting for bicycles, private cars, or walking instead. There was a noted increase in bicycle sales and support for teleworking and online education as ways to reduce car use. Tweets reflected public support for safe reopening policies, such as mask mandates, social distancing, and the expansion of sidewalks and spaces for cyclists. Additionally, users appreciated initiatives that allowed for the use of street spaces for safe commercial activities, such as curbside pickup and outdoor dining, though they also highlighted challenges related to maintaining adequate distancing in high-foot-traffic areas [56,57].

For future emergencies, it would be useful for policymakers to consider what users say about cycling to provide the best service in these circumstances. In addition to the hashtags mentioned above, we find different cities, such as Amsterdam, Copenhagen, and the capitals of two countries, where cycling culture is deeply established [58]. This suggests that people living in these cities and cycling users are accustomed to locating their cities in the posts. This makes it easier to locate posts discussing particular aspects of a city.

### Emojis

The analysis in this study included only emojis, not emoticons. A total of 3,240 unique emojis and emoji combinations were identified. Table 2 presents the most frequently used emojis, including the standard emoji sentiment score, which has been included [59].

**Table 2:** Top 20 emojis found in posts excluding those included in the search equation.

Emoji (sentiment score [−1···+1]) *	Count	Emoji (sentiment score [−1···+1]) *	Count
🚌 Bus (0.190)	6318	💨 Dash symbol (0.381)	2085
🚗 Automobile (0.231)	6109	👏 Clapping hands sign (0.520)	2054
🌳 Deciduous Tree (0.486)	5135	🌲 Evergreen Tree (0.385)	1992
🛴 Kick scooter (--)	4649	👇 White down pointing backhand index (0.247)	1965
🚶 ♀ Pedestrian (--)	4561	🏬 Department store (--)	1931
🚶 Pedestrian (−0.143)	3613	🌄 Sunrise Over mountains (0.125)	1892
👉 Backhand Index Pointing Right (0.390)	3527	🌊 Water wave (0.500)	1850
😊 Smiling Face with Smiling Eyes (0.644)	3366	🚘 Oncoming automobile (0.067)	1836
💜 Red Heart (0.746)	3358	🏙 Cityscape (--)	1820
👍 Thumbs Up (0.521)	3080	😎 Smiling face with sunglasses (0.491)	1793
🏡 House with garden (0.436)	2654	💚 Green Heart (0.656)	1756
➡ Right arrow (0.147)	2451	💪 Flexed biceps (0.555)	1583
☀ Sun (0.465)	2447	🚎 Trolleybus	1577
🏘 Houses (--)	2434	😍 Smiling face with heart-eyes (0.678)	1566
🏛 Classical building	2395	⚡ High voltage sign (0.177)	1524
🏢 Office building (0.154)	2342	🏃 Runner (0.406)	1522
🚍 Oncoming bus (0.071)	2239	✅ Check mark button (0.407)	1443
🚙 Recreational vehicle (0.036)	2223	🎉 Party popper (0.738)	1443
🏠 House building (0.500)	2184	🚕 Taxi (0.000)	1370
🚶 ♂Pedestrian (--)	2122	🌴 Palm tree (0.525)	1357

Sentiment scores are taken from the work by Novak et al. [59] and range from −1–1, with −1 being the most negative possible sentiment and 1 the most positive. The textual meaning has been extracted from the emojipedia database [43].

Among all the emojis found the most repeated was 🚌 (6,318 counts), excluding the ones included in the search equation. This may be because when talking about alternatives to the motor car, the bus is mentioned alongside cycling and walking, for instance:

There's a whole lot of work going on to improve the city right now, so this means there's more traffic on the roads. Why not try an alternative means of transport this week? 🚲 🚌 🚄. https://t.co/Ubl93lrZ4b

The population is aware of alternatives to motor vehicles for getting around cities; however, the use of these sustainable means of transport remains far from the expected level [9]. Thus, new policies must not only provide information about their benefits but also encourage people to use them. Reducing the use of motor vehicles in the city would make it possible to achieve SDG 7 (Affordable and Clean Energy) and 13 (Climate Action).

It should be noted that most emojis had positive sentiment scores. This is the evidence that in the posts analysed, people mostly used emojis with a positive connotation to talk about urban cycling. This is in line with studies that have analysed attitudes towards cycling in urban environments, where opinions are mostly positive [60,61].

Among all the emojis, the pedestrian emoji in its different variants is the one that has been repeated three times, showing that it is a commonly used emoji when users talk about urban cycling. There are active transport promotion publications that offer measures applicable to cycling and walking as a means of transport [62]. This may be because walking is another way of getting around the city in an active lifestyle along with cycling; therefore, they are often proposed as alternatives.

I'm a little concerned that people don't know what to do if gas prices go up. You know there are ways to get around a city without a car right? 🚲 🚶

🚲 🚶 🏃 Best way to really get to know your City.

As we start to reconnect with our wonderful city we’re asking everyone who is able, to consider making at least part of their journey by walking, wheeling or cycling 🚶 ♿ 🚲

Regarding the type of emoji mainly used, we found several related to other means of transport such as buses, automobiles, and kick scooters. Thus, when users comment on aspects of cycling, they include other means of transport, which shows that, in the city, the coexistence of these means of transport in the urban environment is very common. There is scientific literature that analyses the environment of public transport stations and bicycle parking or bike-sharing stations to optimise them to increase their usage [63,64]. Thus, people are likely to discuss these themes in X as follows:

[...] From 🚲 to 🚌, all ages can learn more about what moves people and cities from these titles. https://t.co/wIpG9XCcY9. 📚 #OmahaLibrary #BookList #TransportationBooks

Other recurring emojis have been those related to natural environments such as waves (🌊), different types of trees (🌳, 🌲 or 🌴), a sun (☀) or even a sunrise over mountains (🌄), for instance:

This is what a modern city should look like - everything you need is within a 15 min walk, in your neighbourhood. Less commuting, more living. 🌳 🏃 🚶 👩 🦽 🚴 🏡 https://t.co/SH5tg77ZeC

Stop building roads for cars - build bike lanes and plant trees! 🌳🚴 More trees are the answer to cool down our cities | @guardian: https://t.co/MeUTrDBczt https://t.co/F8P6WW23wn

Have a great holiday weekend everyone! Enjoy the amazing weather and explore our beautiful city! ☀ 🚲 🖒 https://t.co/cWGRTmIMJF

Encouraging more green spaces in the city could promote cycling, and a study revealed that 53.7% of respondents would adapt their cycling route (i.e., use a different route or even accept a longer route) to use green street lanes instead of grey ones [65]. A higher level of green space could also play a positive role in encouraging cycling to and from metro stations, or cycling in general [66]. Therefore, the inclusion of green spaces should be a measure adopted by policymakers to promote cycling as a means of urban transportation. This priority would also coincide with the previously mentioned SDG, in particular ‘Sustainable Cities and Communities’ (Goal 11) in which one of the objectives is to provide universal access to green spaces [67].

### Sentiments scores

**Fig 3:**
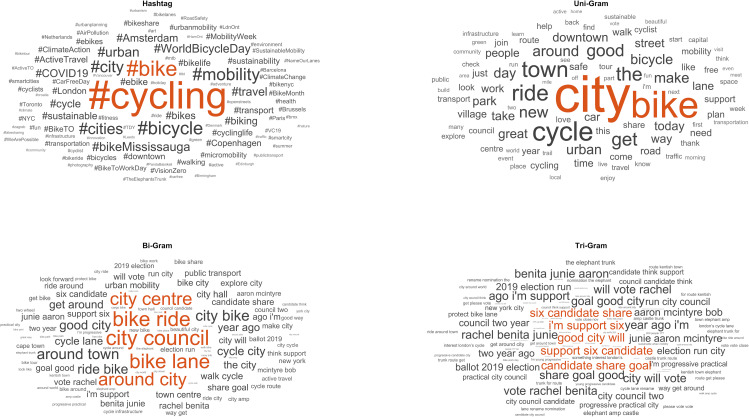
Polarity scores for posts.

The polarity of sentiments showed a mean score of 0.4, where 81,278 of the posts were positive, 25,924 neutral and 8,849 negative (Fig 3). For the analysis of positive posts, we selected those having a polarity score greater than 0.2 (n = 78,011) and for the negative posts a score lesser than 0.2 (n = 6,638). These results show that the majority of people who posted on the subject were positive towards cycling in urban environments; however, this was not the result of previous studies [14]. Nevertheless, the number of positive posts was higher than the number of negative posts, as reported in other articles [15,68].

To further understand user sentiment, we determined the most frequently used words in posts classified as positive or negative. Table 3 lists the top 20 words found in positive and negative posts, excluding those included in the search equation.

**Table 3:** Top 20 words found in positive and negative posts.

Word (positive posts)	Count	Word (negative posts)	Count
good	11047	car	797
great	7631	road	624
new	7383	lane	558
day	6715	stop	558
way	5330	park	526
take	5195	people	525
safe	5146	new	514
today	5115	street	493
park	5085	traffic	462
support	4927	day	426
street	4769	time	422
people	4767	village	357
work	4767	way	356
lane	4701	bad	355
share	4582	today	347
like	4556	work	325
free	4245	centre	311
love	4064	year	310

Top 20 words found in posts classified as positives and negatives, excluding those included in search equation and accompanying verbs or prepositions.

As we can observe, we found words such as ‘car’, ‘road’, ‘lane’, and ‘stop’ related to negative posts. These results indicate a strong concern among bicycle users regarding cycling near cars and roads. Thus, ensuring the safety of urban cycling should be a priority for governments to increase the positive feedback from cyclists to increase cycling. In fact, improving and expanding protected cycling infrastructure is a key issue for increasing cycling in the future [69].

Another recurring word in posts classified as negative has been ‘traffic’. For example, in the following post:

Who said this well said: if you plan cities for cars and traffic, you will get cars and traffic. If you plan for people and places, you will get people and places. Enjoyed cycling 🚲 @urban_innov #KashmirSolidarityDay #cycleIslamabad #ReImagineIslamabad

This is an example of all the comments on X that refer to the elimination of motor vehicle traffic and mixed traffic. Most posts referred to the protection of cycle lanes to prevent traffic from these types of vehicles. Traffic reduction has also been proposed as a measure to reduce pollution, making cities healthier and more sustainable. In this sense, the scientific literature has already made efforts to address the problems generated by motor vehicle traffic alongside urban bicycles. Traffic has emerged as a relevant topic in a review of the scientific literature on urban cycling [70].

Among the posts classified as positive we have found words such as ‘good’, ‘great’, ‘new’, or ‘safe’ [71]. Regarding positive feelings, we found pleasure in cycling, which was probably associated with mental well-being. Cycling influences people’s well-being [72], leading them to experience positive feelings. However, words such as ‘new’ have been found predominantly in negative posts, which may be because the previous studies included only X posts about specific themes such as bike sharing [68].

Other words found in positive posts, such as ‘love’, had been previously identified in research as being among the most mentioned, but ‘free’ was not [15]. This may mean that the sense of freedom that cycling provides in an urban environment is one of the strengths currently valued by the population.

### Main topics found in positive and negative posts

Through an LDA model, the words found were classified into ten clusters, ordered from the highest to lowest probability of occurrence in the entire corpus of posts. Each topic consists of a group of words with an associated probability of appearing together in different posts. The analysis system provides a ranking of the words most related to the topic, as well as a ranking of the posts most likely to pertain to the topic in question. For this research, the most relevant posts (i.e., those with a probability equal to or greater than 0.6) related to the topic have been collected and the most relevant ones have been analysed. Readers who wish to consult the remaining topics can find posts linked to each positive topic in S3 Appendix and each negative topic in S4 Appendix.

**Fig 4:**
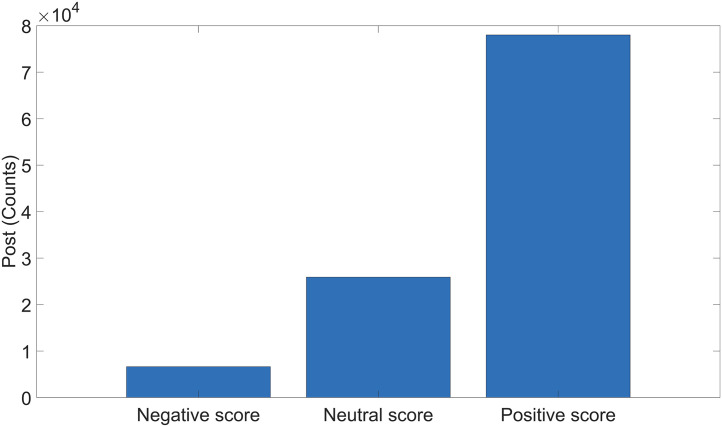
Main topics found in positive posts. Representative words of the topics found in the LDA model are ordered from left to right and from top to bottom according to their normalised values (z-scores). The word size indicates a higher probability of appearing together with the rest of the words in the topic.

Among the topics of the positive posts (Fig 4), topics 1 and 3 refer to mobility and sustainable modes of transport, suggesting that there are issues cycling population identified by their environmental benefits. In this sense, these comments provide evidence that awareness-raising policies work in parts of the population. For example:

New #mobility is influencing how we travel, the places we live and our communities. How do we make sure emerging services + modes are contributing to city policy goals? 🚲 🛴 🛵 🏙 …

However, although this relevant topic indicates that part of the population is aware of the benefits, cycling remains far from what is expected [73]. This emphasises the need for the ongoing implementation of awareness-raising policies aimed at a more significant impact on the population, as current efforts remain inadequate.

Another relevant topic with a positive connotation was related to free-cycling events in urban environments (Topic 2). Among the posts on this topic, we found the promotion of events held in an urban environment where bicycles are the key elements. For example:

Don't forget to come our Dr Bike session, tomorrow, outside the Chiswick Town Hall! Come get your bike repaired for FREE. 🚲 🩺 https://t.co/XGzVmJsdyh

THIS WEEKEND LET'S CELEBRATE ACTIVE SCHOOL TRAVEL! 📣🎉 🚲 [...]

In this context, X has proven to be a common space for promoting cycling events that people consider positively. In this sense, these events have proven successful, as the population has shown a positive view of their celebrations. Another factor to consider is that several events are free of charge. Therefore, those wishing to promote cycling events should note that events with positive impacts are mostly free of charge.

Topic 4 refers to the opportunities offered by cycling to explore a city. Among these posts were the benefits of using bicycles as a means of transportation to visit cities and urban environments. Positive aspects highlighted by users include low cost, outdoor travel, enjoyment of the views and ease of mobility. For instance:

Walking and cycling tours are a great way to explore the city! 🚲 Check out some of our top picks here 👉 https://t.co/hM6AKezjAD #MyOttawa https://t.co/eNLUgrgGSP

The best way to get to know a city is to bike through it! Check out this blog for a self-guided biking tour of GR, featuring classic Grand Rapids stops for you to check out 😇 🚲 https://t.co/mUwMhTeuY2

Cycling, with its remarkable sustainable advantages, is undeniably emerging as a prominent trend in tourism [74]. These results have shown that users consider it a positive experience, it is interesting for cities to promote this type of transport among tourists to attract and encourage sustainable tourism.

**Fig 5:**
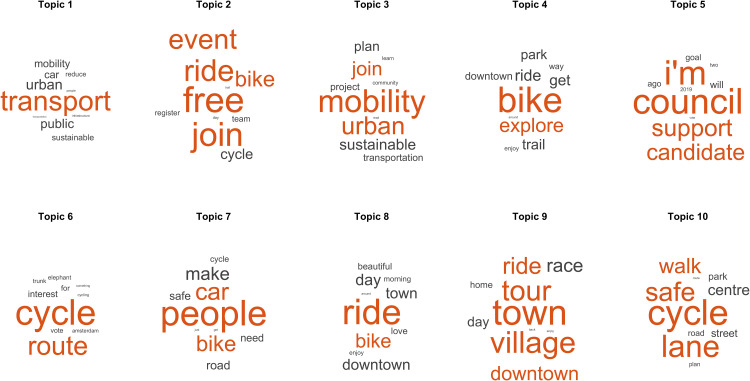
Main topics found in negative posts. Representative words of the topics found in the LDA model are ordered from left to right and from top to bottom according to their normalised values (z-scores). The word size indicates a higher probability of appearing together with the rest of the words in the topic.

As with the positive topics, this study also analyses the negative topics that can be observed in Fig 5. We can see four of the five most relevant topics related to motor vehicles, showing how relevant it is for urban cycling to analyse the coexistence of these modes of transport. Specifically, Topic 4 included comments concerning the sometimes fatal coexistence of bicycles and cars.

Long way to go so that cities, in almost all the world, are more humane and less vehicular. …Rest in peace. 🚲

Another negative topic that emerged was climate change, indicating that comments on X about urban cycling included concerns about replacing cycling with a way to avoid pollution.

#Bicycles are practically #silent. MORE #cycling and LESS #driving massively REDUCES #noisepollution in crowded #cities🚲. Good Monday morning dear #BikeFriends 🙌🏽🚴 ♀ 🚴 ♂ 🚴 💚 https://t.co/OEd6DyoC2h

This means that people are aware of the need to use alternative transport systems to avoid pollution, probably because some policies have been implemented for part of the cycling population. However, although people are aware of how negative motor vehicles can be, their use remains very high across developing countries, and the number of registered vehicles is constantly increasing year by year [75,76]. Policies should be targeted directly at regular users of motor vehicles rather than the general population.

### Analysis based on the post country origins

**Fig 6:**
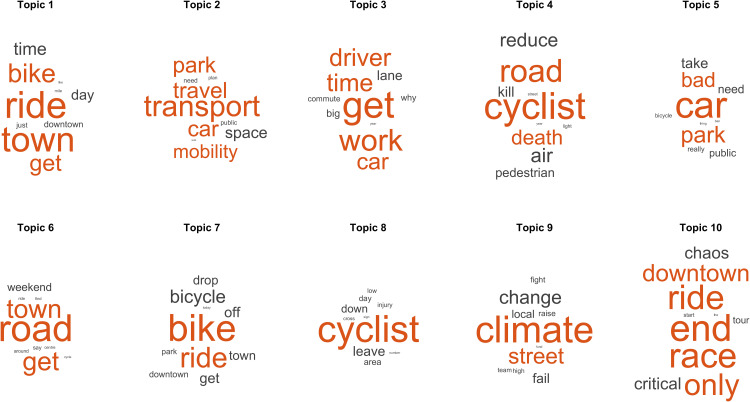
Geolocation of the published posts (n = 10,564).

Concerning the user’s country of origin, the majority were in English-speaking countries, although a notable number were scattered worldwide. The primary country of origin for users was the United States, accounting for 37.93% of the total posts recovered, followed by the United Kingdom (15.86%). Furthermore, the percentage of the authors from highest to lowest included countries, such as Canada, Philippines, Netherlands, Ireland, India, and Australia. The Netherlands has the largest cycling population [77] and a high percentage of English-speaking people [78], although it is not among the countries with the most posts on this subject. Fig 6 illustrates the geolocations of different published posts.

As for the polarity in the feelings of each of the countries, slight differences are observed. In Table 4, the average scores of each of the countries with the most production can be seen. Additionally, the main emojis used in each country are presented.

**Table 4:** Sentiments and emojis based on the country where the post was published.

Country	Count	Positive score	Negative score	emojis
United States	14657	0.211	−0.028	🚲 🚴 🚴 ♀ 🚴 ♂ 🛴 🚗 🚌 🚶 ♀ 🚶 💜
United Kingdom	6868	0.232	−0.033	🚲 🚴 🚴 ♀ 🚴 ♂ 🚘 🚗 🚶 ♀ 🚌 🚶 👍
Canada	2937	0.205	−0.030	🚲 🚴 🔗 🚴 ♀ 🚴 ♂ 🚶 ♀ 🚗 💻 👍 🚌
Kenya	834	0.231	−0.029	🚴 🚲 🚶 👣 🌲 🚴 ♀ 🚴 ♂ 🚸 ♿ 👉
India	809	0.317	−0.020	🚴 🚲 🚴 ♂ 💪 ☺ 🚴 ♀ 🌧 🕺 🚶 🚵
South Africa	777	0.229	−0.022	🚲 🍊 🚴 🚴 ♂ 🚴 ♀ 🧡 💚 📸 🙌 🇿🇦
Australia	464	0.218	−0.039	🚲 🚴 ♀ 🚴 ♂ 🚴 🌏 🚗 ➡ 😎 🚌 🚘
Ireland	276	0.246	−0.025	🚲 🚴 ♀ 🚴 ♂ 🚴 🚶 ♀ 🚗 🌳 😊 🛴 🚌
Spain	178	0.196	−0.023	🚲 🚴 🚴 ♀ 🚴 ♂ 🚌 🚗 👉 😃 🛴 🛵
Switzerland	166	0.298	−0.016	🚲 👇 🚴 ♀ 🚴 ♂ 🚴 🏁 🇩🇰 🌍 💨 📰
Colombia	153	0.223	−0.018	🚲 🚴 ♂ ⛰ 🚴 ♀ 🚴 🏝 🌳 💚 🚴 ♀ 👍
Brazil	125	0.131	−0.128	🚲 🚴 ♀ 🚴 ♂ 🚴 🚳 🚘 ✅ 👏 ⛹ 🌅
Belgium	119	0.239	−0.018	🚲 🚗 🚴 ♀ 🏢 🌳 🚴 🚶 ♀ 🚴 ♂ 🌎 ⬇
Nigeria	116	0.176	−0.037	🚴 🚲 ✈ 🚴 ♂ 🚗 💯 🌍 🌎 🌏 👌
New Zealand	112	0.257	−0.028	🚲 🚴 👉 🚴 ♀ ⛰ 🚴 ‍ ♂ 💚 🚶 ✅ 👍
Nederland	105	0.203	−0.022	🚲 🚴 🚴 ♀ 🇳🇱 🚴 ♂ 🌷 🌳 😍 🚗 🛴
Mexico	86	0.156	−0.008	🚴 ♂ 🚲 🚴 🌱 💯 🚴 ♀ 🤤 🎉 ✨ 🇲🇽
Germany	79	0.217	−0.028	🚲 🚴 ♀ 🚴 ♂ 🌳 🚗 🚴 🏙 😎 🌇 🛴
France	79	0.175	−0.021	🚲 🚴 🚴 ♂ 🚴 ♀ 💜 🙌 🌳 🇫🇷 👉 🚴 ♀
Uganda	70	0.205	−0.046	🚴 🚲 🚴 ♀ 😂 🚴 ♂ 🚶 🚶 ♀ 🌲 🏃 😎
Argentina	57	0.215	−0.014	🚲 💪🏻 🚴 ♂ ✊ 💜 😍 🌳 💪 🚴 🇦🇷
Italy	53	0.163	−0.019	🚲 🚴 ♀ 🚴 🚴 ♂ 👉 ➡ 📷 🌳 💪 😊
Philippines	35	0.152	−0.022	🚲 🚴 🚴 ♀ 🚴 ♂ 🚴 ♂ 🌷 🎸 📌 📷 😁
China	35	0.219	−0.010	🚲 🚴 🚗 ⛵ 🎨 💐 😎 🚴 ♀ 🚴 ♂ ☀
Denmark	33	0.227	−0.042	🚲 🚴 ♂ 🚴 ♀ 🇩🇰 🚴 ⛓ 🌳 👍 😍 ✨
Vietnam	33	0.207	−0.034	🚲 🚴 ♀ 🇻🇳 📸 🚗 🚴 🌃 🍀 🐠 😓
Portugal	29	0.155	−0.021	🚲 🚁 🇵🇹 🌍 🌳 🍃 🎣 💨 📷 🔥
Ecuador	28	0.224	−0.032	🚲 ✨ 🇪🇨 💚 ✅ 👥 🚴 🚴 ♀ 🚴 ♂ 🇳🇱
Turkey	24	0.128	−0.046	🚲 🚴 🚍 🚴 ♂ ⛹ ♀ ✌ 💜 🇳🇱 🏓 💙
Ghana	23	0.295	−0.033	🚴 ♀ 🔻 😘 🚲 🔥 🚴 🏊 ♀ 😒 😳 💜

In Table 5, the main hashtags published in each country can be seen. Unsurprisingly, hashtags like #cycling, #bike, and #bicycle, which are directly related to bicycles, are among the most frequently used. However, concerns about mobility (#mobility) within cities (#city, #cities) also hold a significant presence in most countries. Interestingly, though, hashtags associated with climate change and/or environmental quality are almost entirely absent. Only in Turkey is #CleanAir among the most mentioned hashtags.

**Table 5:** The five most published hashtags in each country.

Country	Top 5 Hashtags
United States	#cycling #bike #bicycle #city #cities
United Kingdom	#cycling #bike #bicycle #Cycling #mobility
Canada	#bikeMississauga #cycling #bike #ActiveTO #bicycle
Kenya	#PandaBaisikeli #cycling #Cycling #COVID19 #mobility
India	#cycling #WorldBicycleDay #bike #city #Cycling
South Africa	#cycling #cities #bicycle #BikeFriends #WorldBicycleDay
Australia	#cycling #bike #liveable #Sydney #betterbybike
Ireland	#cycling #Waterford #mobility #bike #Cycling
Spain	#cycling #bicycle #gijon #valencia #visitgijon
Switzerland	#ZürichCityTriathlon #CyclingForAll #CyclingCities25 #EuropeanMobilityWeek #UCIBikeCity
Colombia	#cycling #Tirana #bike #ColombiaBikeTours #cities
Brazil	#cycling #MobilityWeek #GetToKnowYourDOT #Cycling #micromobility
Belgium	#CohesionPolicy #mobility #cycling #Brussels #SUMP
Nigeria	#cycling #city #cities #mobility #urbanism
New Zealand	#Porirua #DiscoverPorirua #LoveLocal #mountainbiking #MountainBiking
Nederland	#cycling #amsterdam #Amsterdam #city #MobilityWeek
Mexico	#bike #cycling #mobility #Argentina #OnePlanet
Germany	#cycling #bike #WorldBicycleDay #bicycle #BCF
France	#cycling #Paris #hautespyrenees #bike #bicycle
Uganda	#cycling #Kampala #mobility #bike #COVID19
Argentina	#Periscope #GoPro #city #cycling #bike
Italy	#Giro101 #IlikeItaly #Italy #cities #bike
Philippines	#cycling #city #SundayMorning #fitness #amsterdam
China	#cycling #bike #biking #weekend #bikesharing
Denmark	#cycling #bicycle #MobilityWeek #Copenhagen #city
Vietnam	#cycling #bicycle #Vietnam #UrbanPlanning #bike
Portugal	#WorldBicycleDay #Portugal #bike #cities #bicycle
Ecuador	#Quito #cycling #quito #Ecuador #TrueEcuador
Turkey	#cycling #mobility #urban #bicycle #CleanAir
Ghana	#JoySports #Pruride21 #BRT #bike #Cyclothon

### Limitations and future research

Our literature analysis has certain limitations that warrant consideration. It should be noted that our study aimed to provide an overview of the research topic by using text mining techniques, resulting in a broadly generalised discussion. The abundance of topics necessitated a separate analysis in each subject area. This involved exploring specific urban cycling themes on X, akin to the methodologies employed in other studies [68]. Several topics identified in this study could be useful for further research.

We underline that our study has focused on the most frequent and common words that appear in X, as typically occurs when working with large masses of text. Nevertheless, the significance of scientific enquiry may not always align with the terms most repeated or popular among X users. Occasionally, posts incorporate neologisms or words from different knowledge domains, which may be important for cycling in urban environments. Although our study may not have captured fewer posts, they are important and merit further attention. Future studies should explore low-frequency words to identify disruptive or novel ideas that contribute substantially to the research topic. In addition, exploring the texts of other social networks would extend our understanding of the issues under study.

Other interesting lines of work should include searching for X publications in any language using only emojis as search criteria. Subsequently, translation algorithms could be employed to analyze the content, enabling a more universal and inclusive study. Additionally, chatbot-based approaches could be explored, where a chatbot aligned with the Sustainable Development Goals (SDGs) could ask targeted questions about the posts to gain deeper insights into user perspectives. This combination of techniques would help align the research more closely with the SDGs, which aim to represent global perspectives and inclusivity across diverse languages and regions.

Finally, it is necessary to highlight the importance of not inferring conclusions solely based on the visual representation of the figures in this study, as the analysis of the documents classified by our analytical framework offers a greater depth to the interpretation of each one of topic.

## Conclusion

Since the publication of SDGs in 2017, the promotion of cycling as a means of transport has become important to meet these standards. Therefore, promoting cycling as a means of urban transport has become a priority for governments. Moreover, it is important to understand what people think and discuss cycling in urban environments to optimise policy measures. To this end, social media platforms allow users to share opinions and feelings freely and instantly, providing them the opportunity to extract valuable information about an issue using mass-text analysis techniques. This article presents text analysis using text mining methods to explore the most relevant words, hashtags, sentiments, emojis, and topics related to cycling in urban environments on platform X.

Among the most relevant findings of our study, the highly repeated words in the entire text corpus were ‘great’ and ‘good’ — words with a positive connotation. This indicates that the population discussing urban cycling in X had a positive perception. Additionally, ‘bike lines’ and ‘protected bike lanes’ were the most frequently mentioned bigrams and trigrams, respectively. Another aspect analysed was the most-used emojis in posts related to urban cycling. Among these, the most frequently used were emojis representing other means of transport, positive emotions, natural elements, and urban structure. In terms of the number of posts related to the subject, there has been a stabilisation in recent years owing to exponential growth. Moreover, the sentiment attributed to these posts was mostly positive. Concerning the identified topics and frequently reiterated words within them, the positive themes prominently feature the advantages of cycling in urban environments, whereas the negative aspects predominantly revolve around coexistence with motor vehicles.

This article identifies the key issues that the population considers relevant to urban cycling. This enables policies to be oriented towards user concerns and address them, eventually increasing the use of cycling as a means of transport in the urban context. Moreover, including emojis in the search equation allowed us to achieve greater precision in discerning the subject matter of the posts.

## Supporting information

S1 Appendix Raw data. (XLSX)

S2 Appendix n_Grams and hashtag. (XLSX)

S3 Appendix Posts_topic_LDA_positive. (XLSX)

S4 Appendix Posts_topic_LDA_negative. (XLSX)
