# Correction: Trends in the discussion of cycling in urban environments: An X-based study

**DOI:** 10.1371/journal.pone.0342820

**Published:** 2026-05-11

**Authors:** Laura Antón-González, Maite Pellicer-Chenoll, Israel Villarrasa-Sapiña, José-Luis Toca-Herrera, Luis-Millán González, José Devís-Devís

The images for Figs 2–6 are incorrectly switched. The image that appear as Fig 2 should be Fig 3, the image that appear as Fig 3 should be Fig 4, the image that appear as Fig 4 should be Fig 5, the image that appear as Fig 5 should be Fig 6 and the image that appear as Fig 6 should be Fig 2. The figure captions appear in the correct order. The authors have provided a corrected version of figures here.

**Fig 2:**
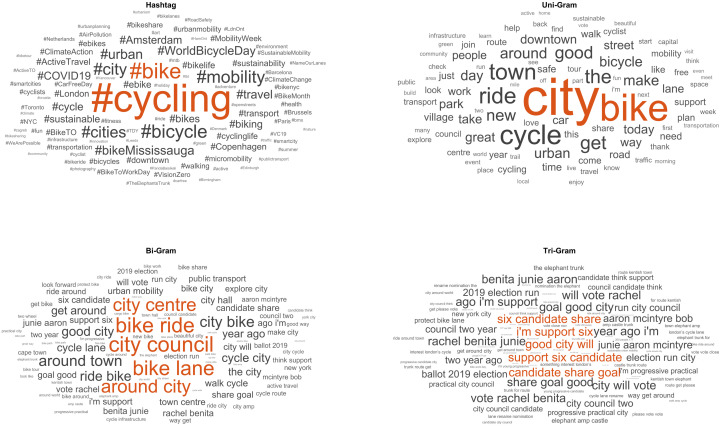
Word clouds of the most frequent words and hashtags found in posts. A larger size represents a higher probability of appearing in the posts and a smaller size represents a lower probability of appearing in the posts. To review terms with lower frequencies of appearance, readers can refer to S2 Appendix.

**Fig 3:**
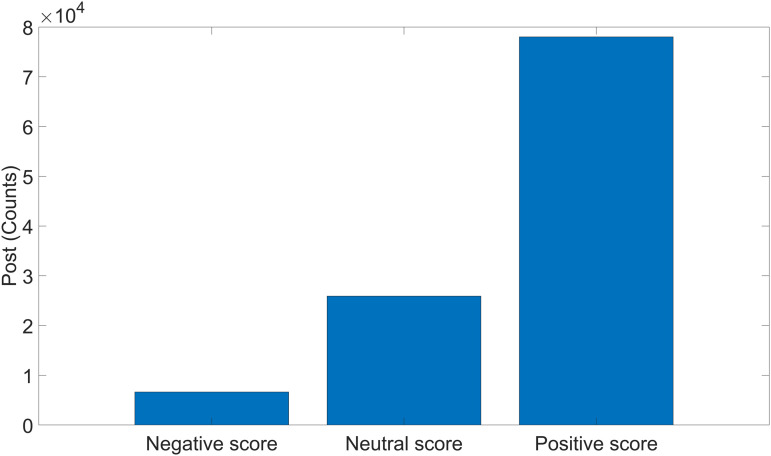
Polarity scores for posts.

**Fig 4:**
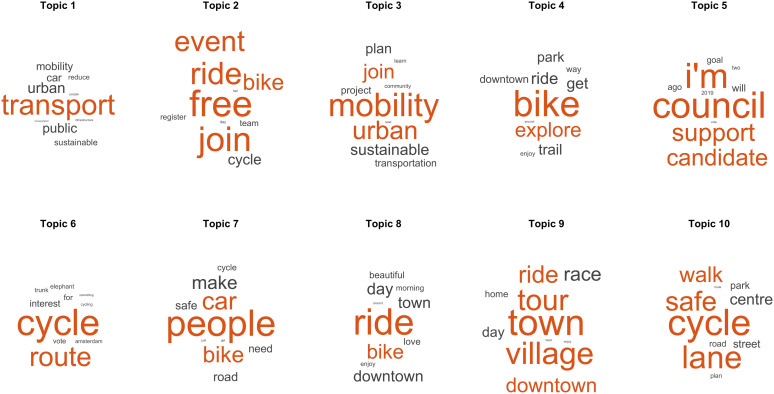
Main topics found in positive posts. Representative words of the topics found in the LDA model are ordered from left to right and from top to bottom according to their normalised values (z-scores). The word size indicates a higher probability of appearing together with the rest of the words in the topic.

**Fig 5:**
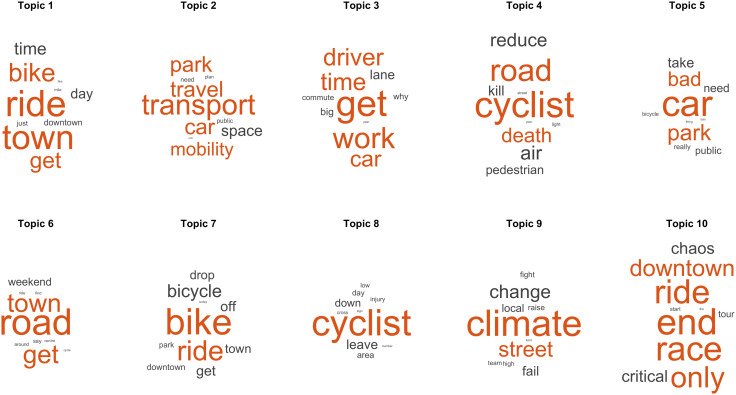
Main topics found in negative posts. Representative words of the topics found in the LDA model are ordered from left to right and from top to bottom according to their normalised values (z-scores). The word size indicates a higher probability of appearing together with the rest of the words in the topic.

**Fig 6:**
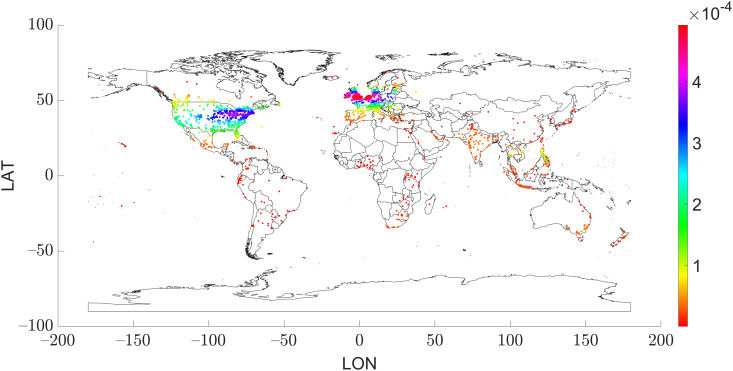
Geolocation of the published posts (n  =  10,564).

## References

[1] Antón-González L, Pellicer-Chenoll M, Villarrasa-Sapiña I, Toca-Herrera J-L, González L-M, Devís-Devís J. Trends in the discussion of cycling in urban environments: An X-based study. PLoS One. 2025;20(11):e0330616. doi: 10.1371/journal.pone.0330616 41252441 PMC12626284

